# Renal dysfunction caused by severe hypothyroidism diagnosed by renal biopsy: a case report

**DOI:** 10.1007/s13730-024-00853-7

**Published:** 2024-02-28

**Authors:** Hiroki Tani, Shuma Hirashio, Akihiro Tsuda, Yoshiro Tachiyama, Shigeo Hara, Takao Masaki

**Affiliations:** 1 Department of Nephrology, National Hospital Organization Hiroshima-Nishi Medical Center, 4‑1‑1 Kuba, Ootake, Hiroshima 739‑0696 Japan; 2 Department of Nephrology, Hiroshima University Hospital, 1-2-3 Kasumi, Minami-ku, Hiroshima 734-8551 Japan; 3 Department of Metabolism, Endocrinology and Molecular Medicine, Osaka Metropolitan University Graduate School of Medicine, Osaka 1-4-3, Asahi-machi, Abeno-ku, Osaka, 545-8585 Japan; 4 Department of Diagnostic Pathology, National Hospital Organization Hiroshima-Nishi Medical Center, 4‑1‑1 Kuba, Ootake, Hiroshima 739‑0696 Japan; 5 Department of Diagnostic Pathology, Kobe City Medical Center General Hospital, 2-1-1 Minatojimaminamimachi, Chuo-ku, Kobe, Hyogo 650-0047 Japan

**Keywords:** Hypothyroidism, Cystatin C, Glomerular filtration rate, Renal biopsy, Renal plasma flow, Abnormal hemodynamic state, Ischemic change

## Abstract

There is a close relationship between thyroid dysfunction and renal dysfunction. However, thyroid dysfunction can unfortunately result in inaccurate measurements of serum creatinine and cystatin C levels. The chronic decrease in cardiac output due to hypothyroidism can reduce renal plasma flow (RPF) resulting in renal dysfunction. We report the case of a 36-year-old male in whom renal dysfunction detected during a company health check-up was found to be caused by severe hypothyroidism. His serum creatinine levels showed poor results, but serum cystatin C levels were within the normal range. The physician thus prioritized serum cystatin C for assessing the patient’s renal function, and concluded that his renal function was normal. He subsequently visited our hospital, aged 36 years, for a comprehensive examination. His serum creatinine level was 1.88 mg/dL and his serum cystatin C level was 0.75 mg/dL, indicating an unusual discrepancy between the two measurements. The patient also presented with fatigue, suggesting hypothyroidism, and we therefore evaluated his thyroid function. His free thyroxine level was below the sensitivity of the assay, while his thyroid-stimulating hormone level was > 100 μIU/mL. A renal biopsy was performed to further explore the underlying cause of his renal dysfunction, which suggested that reduced RPF could be the leading cause of his renal ischemia, with no indications of chronic glomerulonephritis or other abnormalities. His hypothyroidism and renal function improved after thyroid hormone replacement therapy. Given the limited reports of renal biopsy tissue examination during the acute phase of hypothyroidism, the current case provides important information regarding the diagnosis of renal dysfunction in patients with hypothyroidism.

## Introduction

The occurrence of kidney injury, including acute kidney injury, chronic kidney disease (CKD), and nephritis, is usually realized by reduced glomerular filtration rate (GFR) and the presence of urinary disorders, including urinary albumin excretion and/or hematuria. A renal biopsy is considered an important diagnostic method for acute and chronic glomerulonephritis, nephrotic syndrome, rapidly progressive glomerulonephritis, and idiopathic renal disorders. Where possible, renal biopsy is therefore required for a definitive diagnosis of kidney injury in Japan [1].

The assessment of GFR is widely used to provide an estimated GFR (eGFR) using a creatinine-based formula (eGFRcreat); however, serum creatinine is affected by muscle mass, sex, and age [2]. In contrast, cystatin C is not affected by these factors, and serum cystatin C levels and eGFR based on serum cystatin C (eGFRcys) have recently been proposed as better markers of GFR [3, 4]. However, serum cystatin C levels are significantly affected by thyroid dysfunction, making eGFRcys unsuitable for evaluating renal function in patients with thyroid dysfunction [5].

Hypothyroidism is relatively common among patients with CKD who do not require chronic dialysis [6], and hypothyroidism is known to be induced by various mechanisms in CKD [7]. Conversely, primary hypothyroidism has been reported as a risk factor for both cardiovascular events and nephropathy, and hypothyroidism, even in its subclinical form (with mildly elevated TSH), is recognized to contribute to renal functional impairment through mechanisms, such as promoting atherosclerosis and causing direct renal injury [8]. Reduced renal plasma flow (RPF) has also been reported in patients with hypothyroidism.

Although there have been some reports of the use of renal biopsy to diagnose nephritis and nephrotic syndrome induced by thyroid-related antibody deposition on glomeruli [9], reports of the pathological diagnosis of hypothyroidism-induced glomerular ischemia are lacking. In this report, we present the case of a patient in whom dissociation between eGFRcreat and eGFRcys values triggered a diagnosis of hypothyroidism and renal biopsy indicated renal dysfunction caused by ischemia due to hypothyroidism.

## Case report

A 36-year-old Japanese man presented to our hospital for a comprehensive evaluation of his renal dysfunction, which had previously been identified during routine health checks conducted by his employer 3 years earlier. He had no past medical history or family history, and there were no abnormalities noted at birth or during his development. He had received an assessment at the primary care doctor's clinic, including measurements of serum creatinine and cystatin C levels. His serum creatinine level at that time was measured at 1.51 mg/dL (eGFRcreat 45.3 mL/min/1.73 m^2^), indicating stage G3a CKD with renal dysfunction. However, his serum cystatin C level was measured at 0.82 mg/L, with an eGFRcys of 103.5 mL/min/1.73 m^2^. Given that the eGFRcys value was within the normal range, the primary care physician determined that there were no issues with the patient's renal function, and he was accordingly scheduled for regular follow-up, with no additional investigations.

However, further health checks by his employer conducted at age 36 years again indicated renal dysfunction, characterized by elevated serum creatinine levels and a low eGFR. The company consequently instructed him to consult a nephrologist, leading him to seek medical attention at our hospital. Subsequent evaluation at our hospital revealed a serum creatinine level of 1.88 mg/dL, (eGFRcreat 35.1 mL/min/1.73 m^2^) [Table 1]. His renal dysfunction had progressed compared with 3 years previously. However, his serum cystatin C level was 0.75 mg/dL, with an eGFRcys value of 113.2 mL/min/1.73 m^2^, indicating further improvement compared with 3 years ago. These two results showed an unusual discrepancy.

**Table 1:** Laboratory results at the initial visit

Parameter	Value	Reference range
*(Urine)*		
pH	7.5	5.0–6.5
protein	Negative	Negative
Occult blood	Negative	Negative
Red blood cell (/HPF)	< 1	< 5
White blood cell (/HPF)	< 1	< 5
Granular casts (/WF)	Negative	Negative
Urine protein/creatinine ratio (g/g)	0.15	< 0.15
24-h urine protein (g/24 hr)	1.27	< 0.15
*(Blood gas analysis, room air)*		
pH	7.382	7.36–7.44
PaCO_2_	50.3	36–44
HCO_3_^−^	29.2	22–26
*(Blood)*		
Leukocyte count (/μL)	5700	4500–9000
Hemoglobin (g/dL)	13.4	13.6–17.0
Platelet count (× 10^4^/μL)	23.9	14–36
Urea nitrogen (mg/dL)	15.2	8.0–22.0
Creatinine (mg/dL)	1.88	0.60–1.10
eGFRcreat (mL/min/1.73 m^2^)	35.1	< 90
Cystatin C (mg/l)	0.75	0.63–0.95
eGFRcys (mL/min/1.73 m^2^)	113.2	< 90
Uric acid (mg/dL)	9.2	3.6–7.0
Albumin (g/dL)	5.4	4.0–5.0
Aspartate transaminase	138	< 35
Alanine transaminase,	71	< 40
Lactate dehydrogenase (U/L)	402	119–229
Creatine phosphokinase (U/L)	2920	38–196
Sodium (mEq/L)	137	138–146
Potassium (mEq/L)	4.13	3.6–4.9
Chloride (mEq/L)	99	99–109
Corrected serum calcium (mg/dL)	10.3	8.6–10.4
Phosphate (mg/dL)	3.9	2.5–4.7
C-reactive protein (mg/dL)	0.04	< 0.30
Thyroid-stimulating hormone (μIU/ml)	< 100	0.35–4.94
Free triiodothyronine (pg/ml)	< 1.50	1.71–3.71
Free thyroxine (ng/dl)	< 0.40	0.7–1.48
Anit-thyroglobulin antibody (IU/ml)	220.0	< 28
Anti-thyroid peroxidse antibody (IU/ml)	436	0–15

Other physical findings included overall malaise, and blood tests revealed elevated creatine kinase (2920 U/L). In addition, his blood tests showed moderate hepatic dysfunction (Table 1).

**Fig. 1:**
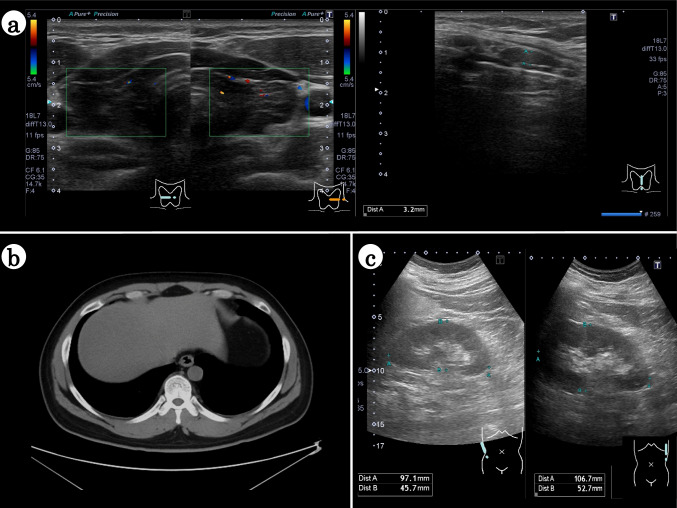
US and CT findings. **a** US images of the thyroid show diffuse swelling and a hypoechoic thyroid. No blood flow signal enhancement was observed. **b** Hematological examination shows liver dysfunction, but liver CT shows no abnormalities in liver morphology. **c** US images of bilateral kidneys show no indications of kidney dysfunction. The patient’s kidneys show a normal morphology and no atrophy. US: ultrasound, CT: computed tomography

On the basis of these findings, we suspected hypothyroidism and conducted a thyroid hormone test. His thyroid-stimulating hormone (TSH) level was > 100 μIU/mL, anti-thyroglobulin antibody titer was 220.0 IU/mL, and anti-thyroid peroxidase antibody level was elevated at 436 IU/mL. Thyroid echocardiography confirmed the presence of a coarse thyroid image (Fig. 1a). The patient was accordingly diagnosed with hypothyroidism attributed to chronic thyroiditis.

Thyroxine and triiodothyronine play vital roles in basal metabolism and thyroid hormones undergo metabolism in the liver. Hypothyroidism has been reported to cause liver dysfunction [10], and the patient’s liver dysfunction was thus attributed to hypothyroidism. Computed tomography revealed no abnormalities in liver morphology (Fig. 1b).

**Fig. 2:**
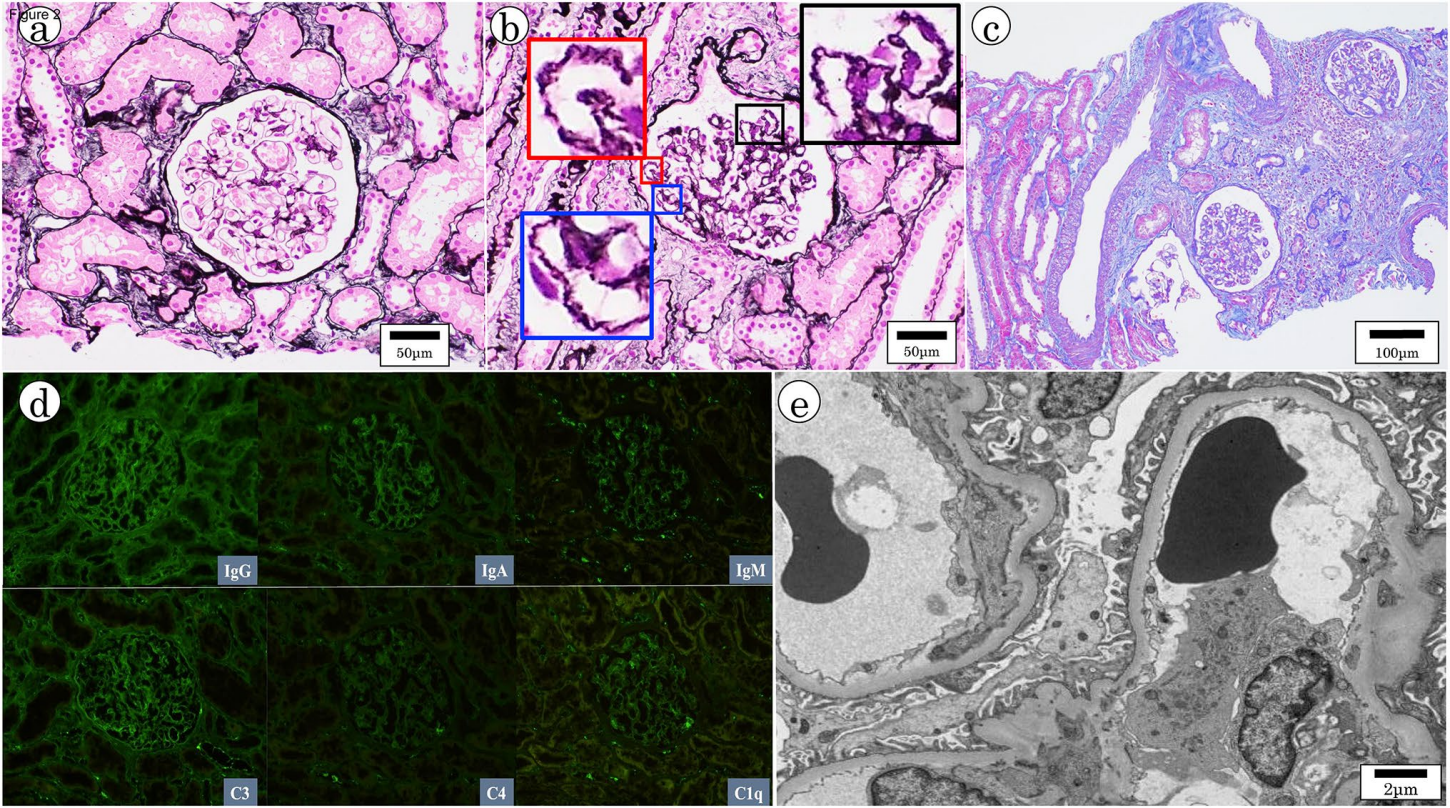
Histological examination of renal biopsy specimens. Periodic acid-methenamine silver staining of renal biopsy tissue (× 400) shows no remarkable findings in most glomeruli (**a**), but wrinkling of the glomerular basement membrane and collapsed glomerular tufts in some glomeruli can be seen (**b**). Black, red, and blue windows in (**b**) represent magnified views of smaller same-colored windows in three locations in the glomerulus. Scale bar = 50 μm. **c** Masson trichrome staining of renal biopsy tissue (× 200) shows focal tubular atrophy and interstitial fibrosis accompanied by mononuclear cell infiltration. Scale bar = 100 μm. **d** Immunofluorescent images of frozen sections are negative for immunoglobulin and complement. **e** Electron microscopy shows intact glomeruli and no organic abnormalities

A renal biopsy was performed to determine if his renal dysfunction was attributable to hypothyroidism. Prior to the renal biopsy, ultrasound examination of his kidneys had revealed no abnormal findings (Fig. 1c). He was admitted to our hospital and underwent three renal tissue sampling procedures from the lower pole of the left kidney using an 18-gage biopsy needle. A total of 70 glomeruli were obtained in the renal biopsy tissue, most of which showed no lesions indicative of glomerular diseases, such as glomerular swelling, basement membrane thickening, mesangial area expansion, or crescent formation (Fig. 2a). However, 29 of 70 glomeruli exhibited segmental or global wrinkling of the glomerular basement membrane (GBM) and a collapsed glomerulocapillary lumen, indicating mild glomerular abnormalities (Fig. 2b). And among the 70 glomeruli, one exhibited global sclerosis. In addition, approximately 15% of the tubulointerstitial area also showed tubular atrophy, mononuclear cell infiltration, and interstitial fibrosis, some arranged in a band-like pattern (Fig. 2c).

Immunofluorescence findings demonstrated no deposition of immunoglobulin or complement in the glomeruli (Fig. 2d). Electron microscopy also revealed no organic changes in the GBM or mesangial areas (Fig. 2e). However, changes such as mild wrinkling were observed in some GBMs.

**Fig. 3:**
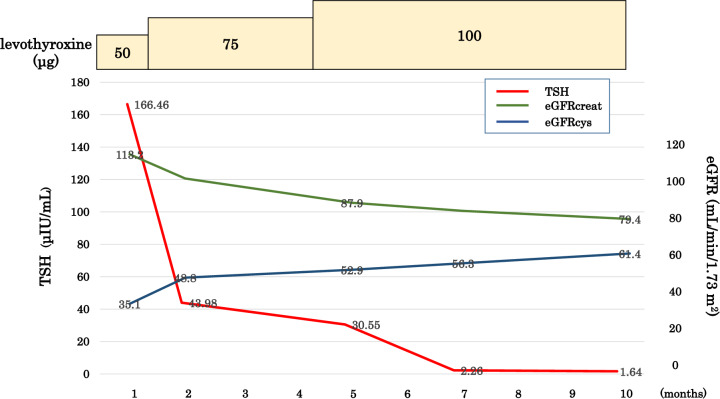
The patient’s clinical course. Changes in the eGFR (eGFRcreat, green lines; eGFRcys, blue lines) and TSH levels (red lines) are shown. The box at the top of the table and the numbers within the box represent the dosage of levothyroxine. TSH: thyroid-stimulating hormone, eGFRcreat: estimated glomerular filtration rate creatinine, eGFRcys: estimated glomerular filtration rate cystatin C

Overall, the findings were interpreted as circulatory-related renal failure, and the primary cause of renal dysfunction was suspected to be influenced by an abnormal hemodynamic state due to hypothyroidism (Fig. 3). The patient’s body mass index was slightly high (29.4 kg/m^2^), but there were no characteristic pathological findings of obesity-related glomerulopathy, such as glomerular enlargement or focal segmental glomerular sclerosis. He had no history of smoking or previous glucose intolerance, and no history of medication ingestion, and there were no identifiable factors suggesting drug-induced chronic tubulointerstitial damage. The case has mild to moderate intimal thickening or mild hyalinosis, which will be consistent with age-related arteriosclerosis, and arteriolosclerosis. We therefore considered that the changes in kidney function and renal biopsy findings were compatible with alterations in hemodynamics due to hypothyroidism. The patient subsequently underwent thyroid hormone replacement therapy. He initially received a daily dose of 50 μg of oral levothyroxine sodium preparation, which was increased to 75 mg at 1-month follow-up, when his TSH level was 166.46 μIU/mL. The treatment was then continued at a dosage of 75 μg for 3 months; however, at that time, his TSH level was 30.55 μIU/mL, leading to a further dose escalation to 100 μg. After 3 months of treatment with 100 μg oral levothyroxine sodium preparation, his TSH level had normalized to 2.26 μIU/mL and his free thyroxine level was 1.35 ng/dL, indicating complete normalization of thyroid function.

After treatment, his serum creatinine level had improved from 1.88 mg/dL (eGFRcreat 35.1 mL/min/1.73 m^2^) to 1.21 mg/dL (eGFRcreat 56.3 mL/min/1.73 m^2^), but his serum cystatin C level had worsened from 0.75 mg/L (eGFRcys 113.2 mL/min/1.73 m^2^) to 1.04 mg/L (eGFRcys 78.5 mL/min/1.73 m^2^). His liver function also normalized.

## Discussion

In the current case, renal biopsy was performed to investigate the patient’s renal dysfunction, despite normal urinalysis findings. Histological findings revealed minimal glomerular abnormalities; however, some glomeruli exhibited wrinkling of the GBM and collapse of the glomerulocapillary lumen, indicating ischemic changes. In addition, in contrast to the expected decrease in eGFRcreat, there was no decline in eGFRcys. Concomitant liver dysfunction, elevated creatinine kinase levels in the blood, and dyslipidemia were also observed, and further investigations revealed the presence of hypothyroidism associated with chronic thyroiditis. Treatment of the hypothyroidism improved the patient’s renal function, evaluated by eGFRcreat, and reduced the dissociation between eGFRcre and eGFRcys.

Patients with CKD are known to have a higher prevalence of thyroid dysfunction compared with the general population [11] due to factors such as decreased renal excretion of iodine [12], increased levels of inflammatory cytokines, and inhibition of thyroid hormone activation caused by oxidative stress [7]. However, the present patient’s renal function was initially within the normal range.

Although hypothyroidism is considered to be a relatively rare cause of renal dysfunction, some nephrologists recognize it as a potential pitfall. The mechanisms of renal dysfunction in hypothyroidism include nephritis, such as membranous nephropathy and IgA nephropathy, which are associated with immunological mechanisms involving the deposition of thyroid peroxidase. However, nephritis was not considered in the present case based on urinalysis and histological findings. On the other hand, reduced RPF has also been reported as a mechanism by which hypothyroidism can cause renal dysfunction, by decreasing cardiac output and direct involvement of renal hemodynamics [8]. Increased creatinine levels in hypothyroidism have previously been suggested to primarily reflect an increase in creatinine production by the muscles, independent of GFR [13]. In contrast however, recent data from humans showed no association between hypothyroidism and urinary creatinine excretion, suggesting that kidney dysfunction truly reflected a decrease in GFR [14]. Accordingly, the current patient showed no increase in urinary creatinine excretion. He also had normal cardiac function, and we therefore suspected that his renal dysfunction was mainly attributable to a reduction in RPF due to direct renal hemodynamic changes caused by thyroid dysfunction.

Although a comprehensive evaluation of the renal artery microcirculation was not feasible in the current case, the presence of renal dysfunction without abnormal urine analysis, histological ischemic finding without organic abnormalities, the absence of other evidence, and the improvement in his renal function following therapeutic intervention for hypothyroidism all supported this mechanism. We were unable to conduct this evaluation because of its excessive invasiveness and a lack of a non-experimental method for accurately evaluating renal microcirculation dynamics. To the best of our knowledge, there have been no reports of renal pathology in hypothyroidism-related renal dysfunction.

Several previous reports also support this potential mechanism. The direct renal hemodynamic effects of hypothyroidism include impaired vasodilation [15], decreased expression of vasodilators, such as vascular endothelial growth factor and insulin-like growth factor-1 [16], increased adenosine [8, 17, 18], and contraction of afferent arterioles [17] due to the tubulo-glomerular feedback mechanism. Pathologically, hypothyroidism has been reported to cause changes in glomerular structure, including thickening of the glomerular basement membrane and expansion of the mesangial matrix, leading to a decrease in RPF [19].

The dissociation of eGFRcreat and eGFRcys and its improvement after treatment for hypothyroidism also may support the involvement of hypothyroidism in the current pathology. Intracellular metabolism is suppressed in patients with hypothyroidism, leading to decreased cystatin C secretion from nucleated cells [20]. Additionally, transforming growth factor beta-1, which stimulates vascular smooth muscle cells to secrete cystatin C [21], is significantly reduced in hypothyroidism. These factors are considered to contribute to the discordance between eGFRcreat and eGFRcys in patients with hypothyroidism.

Thyroid hormones have been reported to exert their effects on thyroid hormone receptors expressed on macrophages, suppressing inflammation and contributing to the inhibition of CKD progression [22]. Furthermore, thyroid hormone replacement therapy may also improve kidney injury due to diabetes [23]. These findings suggest that thyroid hormone replacement therapy is crucial, not only for correcting hormone levels in hypothyroidism, but also for renal protection.

Hypothyroidism is known to be linked to muscle metabolism and may thus affect creatinine values [24]. However, treatment for hypothyroidism in the current patient led to improved renal function within a relatively short period of time. He was a young man and was thus unlikely to experience muscle mass loss within that time frame; furthermore, the renal biopsy findings confirmed the presence of ischemic changes.

Evaluating the microcirculation dynamics of renal arteries in general practice is challenging. To the best of our knowledge, this is the first reported case in which renal dysfunction associated with hypothyroidism-induced ischemia was evaluated through renal biopsy.

The patient was able to avoid irreversible renal dysfunction by receiving thyroid hormone replacement therapy for hypothyroidism. Organ damage, including to the kidneys, caused by changes in hemodynamics triggered by hypothyroidism can be restored through an accurate diagnosis and appropriate treatment.

To demonstrate decreased RPF, it would have been necessary to measure para-aminohippuric acid clearance. In the current case however, consideration of the mechanism of renal dysfunction occurred after confirming the histology of the kidney biopsy and after the initiation of thyroid hormone medication, thus making it unfeasible to perform this measurement. This represents the main limitation of this study.

In conclusion, we present the case of a patient in whom hypothyroidism was identified based on the dissociation between eGFRcreat and eGFRcys, and the presence of renal ischemia was confirmed through renal biopsy. Treatment for hypothyroidism improved his renal function evaluated by eGFRcreat, and reduced the dissociation between eGFRcreat and eGFRcys. This case highlights the importance of considering hypothyroidism as a potential cause of renal dysfunction, and the utility of renal biopsy for elucidating the underlying pathophysiology. The correlation between hypothyroidism and renal dysfunction is significant, emphasizing the importance of thyroid hormone replacement therapy in terms of renal protection.
